# Activated Acinus boosts basal autophagy

**DOI:** 10.4161/23723556.2014.995043

**Published:** 2015-01-23

**Authors:** Nilay Nandi, Lauren K Tyra, Helmut Krämer

**Affiliations:** 1 Department of Neuroscience; UT Southwestern Medical Center; Dallas, TX, USA; 2 Department of Cell Biology; UT Southwestern Medical Center; Dallas, TX, USA

## Abstract

Acinus (Acn) is a nuclear protein that participates in the regulation of autophagy. Loss of Acn function prevents autophagy in starving cells. Conversely, Acn activation induces basal autophagy. This enhances the quality control functions of autophagy such as the removal of misfolded proteins, thereby reducing neurodegeneration and prolonging lifespan. Acn activity is enhanced by Akt1-mediated phosphorylation, which counteracts the cleavage of Acn by a caspase-3 homolog.

Macro-autophagy (here simply referred to as “autophagy”) is a catabolic process that helps cells cope with starvation by non-specifically degrading cellular content to reclaim necessary nutrients. In a second role, autophagy operating at basal levels contributes to quality control in cells and aids in the removal of aberrant proteins or organelles that can accumulate in response to a variety of cellular stresses. It is important to understand how the basal level of autophagy is adjusted to varying degrees of stress in the absence of starvation as failures in quality control have been linked to neurodegenerative diseases, inflammation, and cancer.^1^

Our recent work^2^ indicates that Acinus (Acn) protein may play a role in that regulation. Acn is a conserved nuclear protein that interacts with the exon junction complex and spliceosomes and thereby modulates specific RNA splicing events^3, 4^. The first indication of a role in autophagy came from *acn* loss-of-function mutations, which interfere with autophagosome maturation.^5^ In well-fed *Drosophila* autophagy is inhibited by target of rapamycin (TOR) signaling,^6^ but this inhibition can be bypassed by Acn overexpression. Even more strikingly, high-level Acn expression is lethal, but viability is restored when autophagy is blocked.^5^ These observations emphasize the importance of the control of Acn activity for adjusting the levels of autophagy. In our recent study^2^ we explored how multiple intracellular signals can modulate Acn levels, which might serve as a focal point for adjusting the basal level of autophagy.

The dynamic regulation of Acn is exemplified in the developing eye where its expression in photoreceptor cells changes rapidly depending on cell type and developmental stage.^5^ To our surprise, a GFP reporter transgene detected little regulation at the transcriptional level; instead, Acn is primarily regulated at the level of protein stability.^2^ Two conserved elements that contribute to its regulation are a caspase cleavage site^7^ and a pair of Akt1 consensus target sites.^8^ To test their importance *in vivo* we generated transgenic flies in which endogenous Acn was replaced by mutant proteins designed to prevent cleavage by caspases (Acn^D527A^*)* or mimic phosphorylation at the Akt1 target sites (Acn^S641,731D^). Both of these mutations stabilized Acn and yielded interesting physiological consequences.

In these mutant animals, all stages of autophagy were enhanced, whether evaluated by live imaging of GFP-Atg8a fluorescence in early autophagosomes, antibody staining for endogenous Autophagy-related 8a (Atg8a) protein, or lysotracker accumulation in autolysosomes. Electron microscopy revealed a remarkable increase in size and abundance of autolysosomes compared to control animals. The relative increase in autophagy was most pronounced in fed animals, indicating that elevated Acn levels enhanced basal, starvation-independent levels of autophagy. The resulting improved cellular quality control was evident from an enhanced ability to clear aggregation-prone poly-Q Huntingtin protein from photoreceptors and, maybe most strikingly, a significantly prolonged longevity (**Fig. 1**).

**Figure 1.**
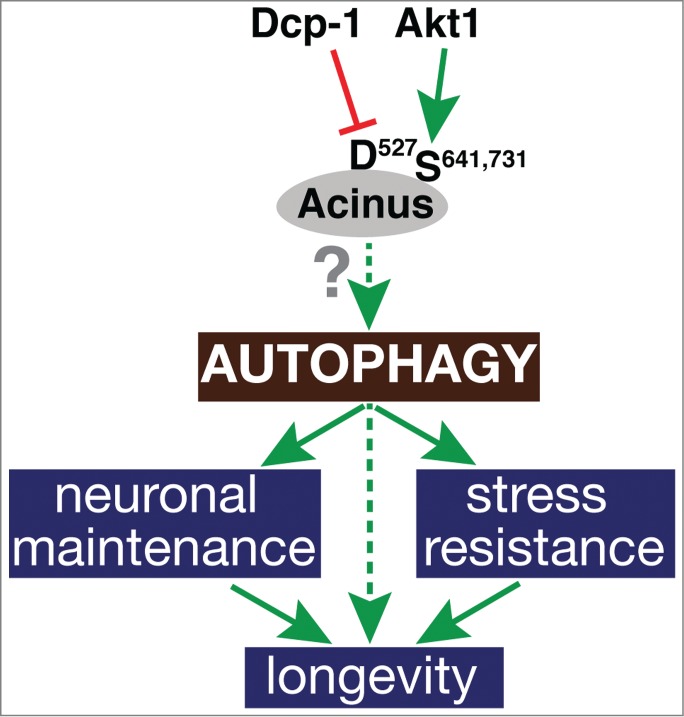
Benefits of elevated Acinus activity. Acinus levels are controlled by the combined activities of death caspase-1 (Dcp-1) and Akt1. Elevated Acinus activity enhances basal autophagy, and thereby improves stress resistance, neuronal maintenance, and, ultimately, longevity of flies.

To identify the protease responsible for Acn cleavage we used a targeted RNAi screen. Acn overexpression in the eye causes mild eye roughness that served as readout. Among the 9 *Drosophila* caspases tested in this screen only knockdown of death caspase-1 (Dcp-1), a homolog of mammalian caspase-3, enhanced Acn function. Elevated levels of Acn in *dcp-1* mutant cells and the resulting upregulation of autophagy substantiated the physiological relevance of the genetic interactions in the eye.

Sahara and colleagues^7^ first detected cleavage of mammalian Acn proteins by caspase-3 activity that was upregulated in the context of apoptotic cells. Our observations extend their original finding to cells in different developing tissues, including precursors of photoreceptors and fat body cells. These healthy, non-apoptotic cells use their low background levels of caspases to inactivate Acn as they balance the beneficial effects of quality control autophagy with the deleterious effects resulting from its overactivation. Acn may not be the only target in such contexts, as cleavage of Beclin-1 and autophagy-related 4 (Atg4) by caspases can also reduce autophagy and suppress its cytoprotective function.^1^

Regulated cleavage of Acn by Dcp-1 may also be the key to understanding the genetic interactions between Akt1 and Acn. Their co-expression worsened eye roughness induced by wild-type Acn, but not by Acn mutants lacking the Akt1 target sites. Consistent with the effect of *in vitro* phosphorylation of mammalian Acn by Akt1,^8^ we found that *Akt1* knockdown in *Drosophila* reduced Acn phosphorylation *in vivo* and prevented its protective function against Acn cleavage.

Surprisingly, Akt1-mediated stabilization of Acn and the resulting upregulation of basal autophagy appear to be opposite to the well-documented role of Akt1 as a negative regulator of autophagy in the context of the canonical PtdIns3K–Akt–TOR pathway.^1^ One possible explanation for this contradiction could be the existence of distinct pools of Akt1.^9^ A pool of cytoplasmic Akt1 may inhibit autophagy by phosphorylating the tuberous sclerosis complex 2 (TSC2) protein and Beclin.^1,6^ In contrast, we speculate that a nuclear pool of Akt1 may phosphorylate Acn, and possibly other nuclear targets yet to be identified, leading to increased basal autophagy.

How Acn induces basal autophagy remains unclear. As Acn is primarily nuclear and part of the apoptosis-and splicing-associated protein (ASAP) complex, one possible mechanism may relate to the regulated splicing of genes involved in autophagy. Alternatively, we detected cytosolic Acn in a subset of cells in developing eyes, raising the possibility that Acn functions outside the nucleus.^5^ Deciphering the mechanisms by which Acn regulates basal autophagy will be an important goal of our future research.
